# The Relationship Between Therapist Effects and Therapy Delivery Factors: Therapy Modality, Dosage, and Non-completion

**DOI:** 10.1007/s10488-016-0750-5

**Published:** 2016-07-16

**Authors:** David Saxon, Nick Firth, Michael Barkham

**Affiliations:** 1 Centre for Psychological Services Research, University of Sheffield, ScHARR, Regent Court, Regent St., Sheffield, S1 4DA UK; 2 School of Health and Related Research, University of Sheffield, ScHARR, Regent Court, Regent St., Sheffield, S1 4DA UK

**Keywords:** Therapist effect, Variability, Depression, Drop-out, Dose effect

## Abstract

To consider the relationships between, therapist variability, therapy modality, therapeutic dose and therapy ending type and assess their effects on the variability of patient outcomes. Multilevel modeling was used to analyse a large sample of routinely collected data. Model residuals identified more and less effective therapists, controlling for case-mix. After controlling for case mix, 5.8 % of the variance in outcome was due to therapists. More sessions generally improved outcomes, by about half a point on the PHQ-9 for each additional session, while non-completion of therapy reduced the amount of pre-post change by six points. Therapy modality had little effect on outcome. Patient and service outcomes may be improved by greater focus on the variability between therapists and in keeping patients in therapy to completion.

## Introduction

The past 50 years has seen a concerted effort by researchers to develop more effective models of therapy. The dominant research method for testing the efficacy of such models has been the randomised controlled trial (RCT) and results have been summarised by national policy bodies [e.g., Substance Abuse and Mental Health Services Administration (SAMDSA), National Institute for Health and Care Excellence (NICE)] to support the adoption of efficacious, evidence-based treatments into routine clinical practice. For example, the Australian Department of Health requires Medicare-funded treatments to be evidence-based (Department of Health 2012), and treatment provision decisions made by the American Medicare and Medicaid governmental programs are influenced by the AHRQ (Agency for Healthcare Research and Quality 2002).

In the UK, NICE (2016a) policy guidelines are used by the UK Department of Health to decide which treatments are to be funded by the National Health Service. For depression in adults, NICE guidelines recommend Cognitive Behaviour Therapy (CBT) as the most effective therapy model, although inter-personal therapy (IPT) and to a lesser extent, counselling are also supported (NICE 2016b). The guidelines note that although provision of the latter gives patients more choice, there is greater uncertainty about its effectiveness (NICE 2016b).

In contrast to research into therapy models, there has been relatively little research into the variability between the *therapists* providing the therapy, despite therapists representing a large resource (as well as cost) in clinical settings. The phenomenon of therapist variability is termed the *therapist effect*. In RCTs designed to compare therapy models, such variability is often constrained by therapist selection, training, supervision and close monitoring of protocol adherence. Also, to reliably estimate the size of therapist effects a large sample of therapists and a very large sample of patients are required (e.g., Maas and Hox 2004; Soldz 2006), which can be problematic for RCTs. However, underestimating or ignoring therapist effects risks overstating the effect of the therapy model (Kim et al. 2006). In order to estimate therapist effects, researchers have focused on large samples of routinely collected data from clinical practice (Elkin et al. 2006; Lambert and Okiishi 1997; Soldz 2006). The study of these large datasets, to consider patient outcomes in ‘real world’ settings, has been termed practice-based evidence (see Barkham et al. 2010; Castonguay et al. 2013).

Accumulating evidence from both trials and routine data has shown that therapists have a significant effect on patient outcome. Results indicate that therapists account for around 5–10 % of unexplained variance in patient outcomes, with 8–9 % being most commonly reported. These results hold in different therapy models and after controlling for confounding patient variables (Crits-Christoph et al. 1991; Crits-Christoph and Mintz 1991; Kim et al. 2006).

There has been little research into why some therapists are more effective than others, even when delivering the same therapy model and controlling for case-mix. Therapist factors such as training, skill and experience (Beutler et al. 2004) and adherence to treatment protocol (Webb et al. 2010), have been found to be only weak predictors of patient outcome. The strength of the therapeutic alliance has been shown to be a stronger predictor (e.g. Arnow et al. 2013; Falkenström et al. 2013), with evidence indicating that therapists vary in their ability to recognise and repair ruptures to that alliance (Safran and Muran 2000).

In addition to studies of therapy models and therapist effects, there is a growing body of evidence focusing on variables involved in the *implementation* of psychological therapy. Therapeutic “dose” (number of sessions received) and non-completion (unilateral termination of therapy by the patient, often termed “dropout”) have seen particular research interest.

Therapeutic dose has been found to be related to more desirable clinical outcome and policy guidelines often suggest optimum treatment lengths. For example, NICE guidelines suggest 16–20 sessions of CBT for depression (NICE 2016b). However, in practice most patients receive fewer sessions, with 6 sessions of CBT being the average in primary care in the UK (Health & Social Care Information Centre 2014). Further, the precise relationship between dose and outcome has been contentious (Baldwin et al. 2009; Barkham et al. 2006; Howard et al. 1986) and an important question for policymakers and services is “how much is enough”?

Non-completion similarly remains an important issue despite decades of research (Barrett et al. 2008). Large-scale studies show that patients do not complete around 20–35 % of psychological therapy interventions (Cooper and Conklin 2015; Hans and Hiller 2013; Roos and Werbart 2013; Royal College of Psychiatrists 2013; Swift and Greenberg 2012). Therapy non-completion greatly impedes effective therapy delivery across treatment modalities, contexts and patient populations (Barrett et al. 2008), and is associated with poorer clinical outcomes (Cahill et al. 2003). Research has indicated that therapist factors such as skill and experience, a weaker therapeutic alliance and fewer attended sessions are associated with increased therapy non-completion (Fernandez et al. 2015; Roos and Werbart 2013).

Given the significance of therapist effects and the importance of delivery factors such as therapeutic dose and non-completion of therapy to patient outcomes, the current study used a large sample of routinely collected data, to consider how the variability between therapists outcomes relates to the number of sessions patients attended and whether they dropped out of therapy or not. As the sample contained data from both CBT therapists and counsellors, the variability in outcomes due to therapy model was also considered.

Accordingly, the aim of the study was to use multilevel modeling to estimate the size of therapist effect, controlling for case-mix, then assess the variability in therapist effectiveness in relation to: (1) treatment modality, CBT or counseling; (2) therapeutic dose, the number of sessions attended, and (3) treatment ending, completion or non-completion.

## Methods

### Study Setting

The context for the present study is the UK government’s Improving Access to Psychological Therapies (IAPT) initiative. IAPT aims to provide evidence-based psychological interventions for common mental health problems in primary care. In accordance with NICE guidelines (National Institute for Health and Care Excellence 2011), IAPT uses a stepped care therapy delivery model (CSIP Choice and Access Team 2008), delivering high-intensity psychological therapies, mainly cognitive behaviour therapy (CBT) and counseling, at step 3.

### Original Dataset

The initial data set comprised 39,520 patients who attended the service from June 2010 to October 2013. The service provides primary care psychological therapies at around 90 GP practices across a city with a population of around 550,000. In line with IAPT services nationally, the service offers a stepped care model of care with the vast majority of patients being offered a low intensity treatment at step 2, such as guided self-help, computerised CBT and educational groups. Patients with depression who are stepped–up to step 3 are generally offered 8–12 sessions of one-to-one therapy, either CBT or counseling, with the option to extend to 20 sessions if necessary. The data collected by the service conforms to the standardised IAPT minimum dataset (IAPT MDS) and includes patient demographic information, outcome measures and information about the treatment in terms of therapy type, number of sessions attended and type of treatment ending. Ethical approval for the current study was granted by the regional ethics committee (16/YH/0028).

### Study-Specific Data Set

Most patients (N = 25,619) received a step 2 treatment and were excluded, as were patients who received other therapies (e.g., couples and family therapy, behavioural activation). The service does not carry out formal diagnoses, but patients were included in the current study if they scored above the clinical cut-off on a standardized outcome measure of depression (see later). Patients were included if they received between two and 20 sessions of one-to-one therapy (counselling or CBT), and completed a common standardised outcome measure at the first and last session of treatment. Further, to improve the reliability of parameter estimates only therapists with 20 or more patients were included (Schiefele et al. in press).

The resulting dataset comprised 4034 patients [CBT: 1912 (47.4 %); Counseling: 2122 (52.6 %)] seen by 61 therapists (28 CBT, 33 counsellors). The mean (SD) age of patients in the study sample was 42.1 (13.77) years, 70.1 % were female, 90.0 % were white and 33.0 % were unemployed.

## Measurement: Assessment and Outcome

Our primary measure was the Patient Health Questionnaire-9 (PHQ-9; Kroenke et al. 2001). The PHQ-9 is a nine item measure of depression. Each item is rated from 0 to 3. Scores can range from 0 to 27, with higher scores indicating more symptoms of depression. The primary outcome was the pre-post change on the PHQ-9. Therefore, positive values were indicative of patient symptom improvement, whilst negative values indicated that their symptoms had worsened.

In a primary care population, the PHQ-9 has demonstrated good internal validity (Cronbach’s α = 0.89), test–retest reliability (0.84 intraclass correlation), and sensitivity and specificity (each 0.88 using a clinical threshold of 10) (Kroenke et al. 2001). The PHQ-9’s validity is supported in general and primary care populations (Cameron et al. 2008; Martin et al. 2006), and it correlates highly with the Beck Depression Inventory and 12-item General Health Questionnaire (Martin et al. 2006). Although measures were completed sessionally, the service could only provide the first and final (pre and post) recorded scores. This meant that although a final measure was available for both therapy completers and drop-outs, the actual trajectories of change during the course of therapy could not be analysed. Instead, we produced a simple measure of ‘average change per session’, by dividing the amount of pre-post change by the number of sessions attended.

To determine statistically reliable and clinically significant improvement (i.e. ‘recovery’) rates, we adopted the procedures as set out by Jacobson and Truax (1991)—that is, the change scores for patients had to be greater than the reliable change index in order to take account of measurement error, and the end point score had to move from above the cut-off level to below this predetermined score. For the PHQ-9, we used a cut-off score of 10 and a reliable change index of 6 points (McMillan et al. 2010).

In order to compare therapist outcomes, significant case-mix variables need to be controlled for in the analysis. Variables available, in addition to intake PHQ-9 score, were patient demographic variables, age, gender, ethnicity and employment status and severity of anxiety at intake, as measured by GAD-7 (Spitzer et al. 2006).

### Analysis

The statistical concepts and methodology of MLM are fully described elsewhere (e.g., Rasbash et al. 2009b; Raudenbush and Bryk 2002; Snijders and Bosker 2012). A single level regression model containing explanatory patient variables, with continuous variables grand mean centered (Hofmann and Gavin 1998; Wampold and Brown 2005), was developed. Explanatory variables were tested for significance by dividing the derived coefficients by their standard errors with values greater than 1.96 considered significant at the 5 % level. The single level model was extended to a multilevel model allowing the variance in patient outcome to be split between the patient level (level 1) and the therapist level (level 2).

Multilevel modeling software MLwiN v2.30 (Rasbash et al. 2009a) was used to estimate parameters, using Iterative Generalised Least Squares (IGLS) procedures. Whether the multilevel model was a better fit for the data than the single level model, and whether there was a significant therapist effect, were tested by comparing the difference in −2*loglikelihood ratios produced by the single and multilevel models, against the chi squared distribution for the degrees of freedom of the additional parameters. Variability between therapists in the relationship between each explanatory and outcome variable was considered using random slope models.

The size of the therapist effect is the proportion of the total variance that is at the therapist level (level 2; Wampold and Brown 2005). This therapist effect is the amount of variability in patient outcomes that is attributable to unexplained differences between therapists, after controlling for variables in the model (i.e., controlling for case-mix).

The therapist residuals produced by the model represent the degree to which each therapist varies in their impact on outcomes, relative to the average therapist. Positively signed therapist residuals are associated with increasing outcome scores (i.e. greater pre-post change), while negatively signed residuals are associated with a reduction in outcome score (i.e. less pre-post change). The size of the residuals can therefore be used to make comparisons between therapists (Goldstein and Spiegelhalter 1996; Saxon and Barkham 2012).

The therapist residuals are assumed to have a normal distribution and a mean of zero. By ranking and plotting the residuals with their 95 % confidence intervals (CIs), three groups of therapists were identifiable. Therapists whose CIs crossed the average residual (zero), were not considered significantly different to the average therapist. Therapists whose CIs did not cross zero were considered either significantly above or below average in their effect on patient outcomes.

Following the development of the model containing case-mix variables, our variables of interest, treatment modality (as a therapist level variable), dosage and ending type, were added to the model. Those found to be significant predictors of outcome were then considered in relation to the three groups of therapists, average, below average and above average, identified above.

## Results

### Multilevel Model

The multilevel model was developed from a single level regression model that included significant patient predictors of pre-post change on the PHQ-9. A comparison of the −2*loglikelihood ratios of the two models showed a significant reduction when the effect of the therapist was allowed to vary (*χ* ^2^(1) = 90.89, *p* < 0.001), indicating that the multilevel model was a better fit for the data and there was a significant therapist effect. Consideration of the quartile–quartile plots of the patient and therapist residuals indicated that Normality can be assumed. (The multilevel model is presented in “Appendix”).

The negative coefficients in the model show that being unemployed, non-white, or having greater intake severity on GAD-7 reduced the amount of pre-post change on PHQ-9. Higher intake scores on PHQ-9 were predictive of greater improvement. However, this may be in part a statistical function in that higher PHQ-9 scores have more scope to improve. There was also a significant interaction between employment status and PHQ-9 intake score, with unemployed patients who had higher PHQ-9 scores at intake making less change than employed patients with similar levels of severity.

### Therapist Effect

The model indicates the intercept (average therapist pre-post change) to be 7.847 with a variance (SE) of 2.117 (0.499). This is the therapist level variance. The variance (SE) at the patient level is 34.641 (0.777), giving a total variance of 36.758, of which 5.8 % is at the therapist level. This therapist effect of 5.8 % represents the amount of variability in patient outcomes attributable to therapists.

### Therapist Residuals

**Fig. 1:**
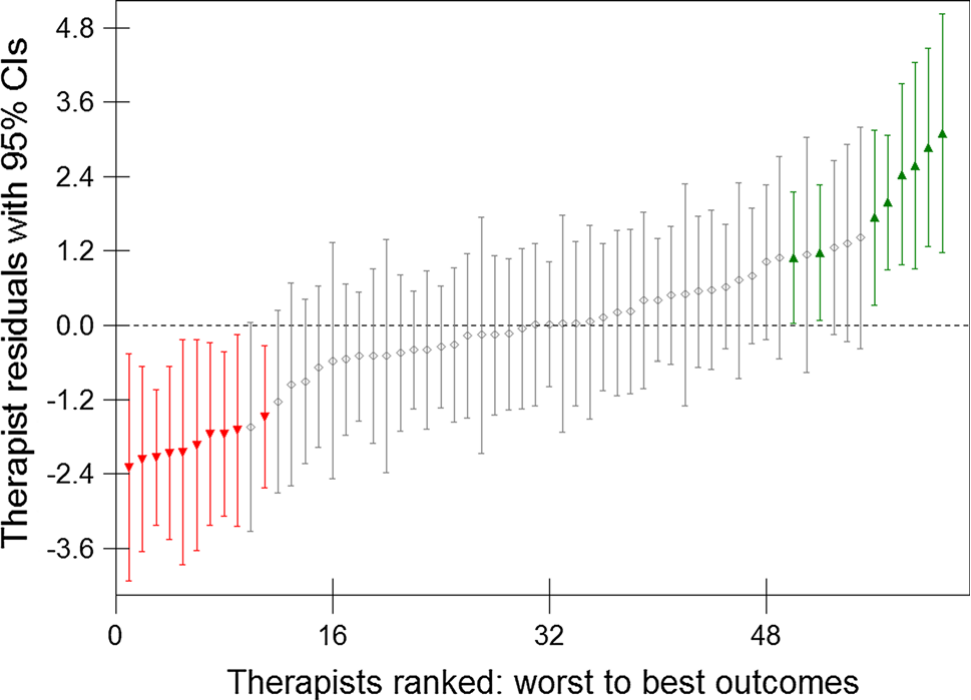
Ranked therapist residuals produced by the model, with 95 % confidence intervals (CIs)

Figure 1 illustrates the variability between therapists by ranking and plotting the therapist residuals (*u*_0j_) produced by the model with their 95 % confidence intervals. The ‘average’ therapist is represented by the dashed horizontal line, where the residual equals zero, Therapists whose confidence intervals do not cross zero are significantly below average, highlighted on the left of the plot (N = 10), or significantly above average, highlighted on the right of the plot (N = 8). Most therapists (N = 43) were not significantly different from the ‘average’ therapist.

### Therapist Outcomes

Overall, the mean (SD) patient PHQ-9 score at intake was 17.2 (4.48), while the mean (SD) PHQ-9 score at the last attended session was 10.4 (6.93) with a mean (SD) pre-post change of 6.8 (6.33) points. The amount of patient change ranged from −15 to 27 points, and 45.2 % of patients made statistically reliable and clinically significant improvement.

Table 1 describes the clinical outcomes of the three groups of therapists identified in Fig. 1 and shows above average therapists to be over twice as effective as below average therapists, with a mean (SD) pre-post change of 9.9 (1.65) points on the PHQ-9 and a mean (SD) recovery rate of 63.7 % (9.69) compared with 4.2 (0.93) points and 25.6 % (6.43). The bulk of therapists had outcomes similar to the overall patient outcomes above, with a mean pre-post change (SD) of 6.8 (0.96) points and mean (SD) recovery rate of 46.4 % (9.86). The non-overlapping ranges of therapist outcomes for below and above average therapists suggest that the model has identified two distinct groups in terms of their outcomes.

**Table 1:** Outcomes for average and above and below average therapists identified by the model

	Therapist group		
	Below average	Average	Above average
N (%) therapists	10 (16.4)	43 (70.5)	8 (13.1)
N (%) patients	543 (13.5)	2958 (73.3)	533 (13.2)
Therapists pre-post change mean (SD)	4.2 (0.93)	6.8 (0.96)	9.9 (1.65)
Therapist pre-post change range	2.7–5.3	4.6–9.1	7.9–12.7
Mean (SD) recovery rate	25.6 (6.43)	46.4 (9.86)	63.7 (9.69)
Recovery rate range	16.0–37.1	21.9–71.4	49.6–75.8

### Therapy Modality

Comparing raw patient outcomes between the two modalities, CBT showed more pre-post change than counseling, with a mean (SD) change of 7.3 (6.35) points compared with 6.3 (6.28) points, giving a small effect size (Cohen’s *d*) in favour of CBT of 0.16. Therapy type was also significant when added to the multilevel model, with counselling producing 0.8 of a point less improvement than CBT after controlling for other variables (coefficient: −0.84; SE: 0.41).

Patients receiving counseling were more likely to complete therapy, with a non-completion rate of 29.4 % compared with 33.4 % for CBT (χ^2^(1) = 7.72, *p* = 0.005), and tended to have fewer sessions. Patients receiving counselling had a mean (SD) of 6.1 (3.56) (*Median*: 5) sessions, compared with a mean (SD) of 8.1 (4.74) (*Median*: 8) sessions for CBT (M-W U Test: *p* < 0.001).

When sessions attended and ‘therapy ending’ were added to the model, and the effect of either was allowed to vary between individual therapists (using random slopes), modality was no longer significant. This suggests that the variability between individual therapists is more important than the variability between the therapy types in the relationships between dose and outcome and ending and outcome.

### Therapeutic Dose

**Fig. 2:**
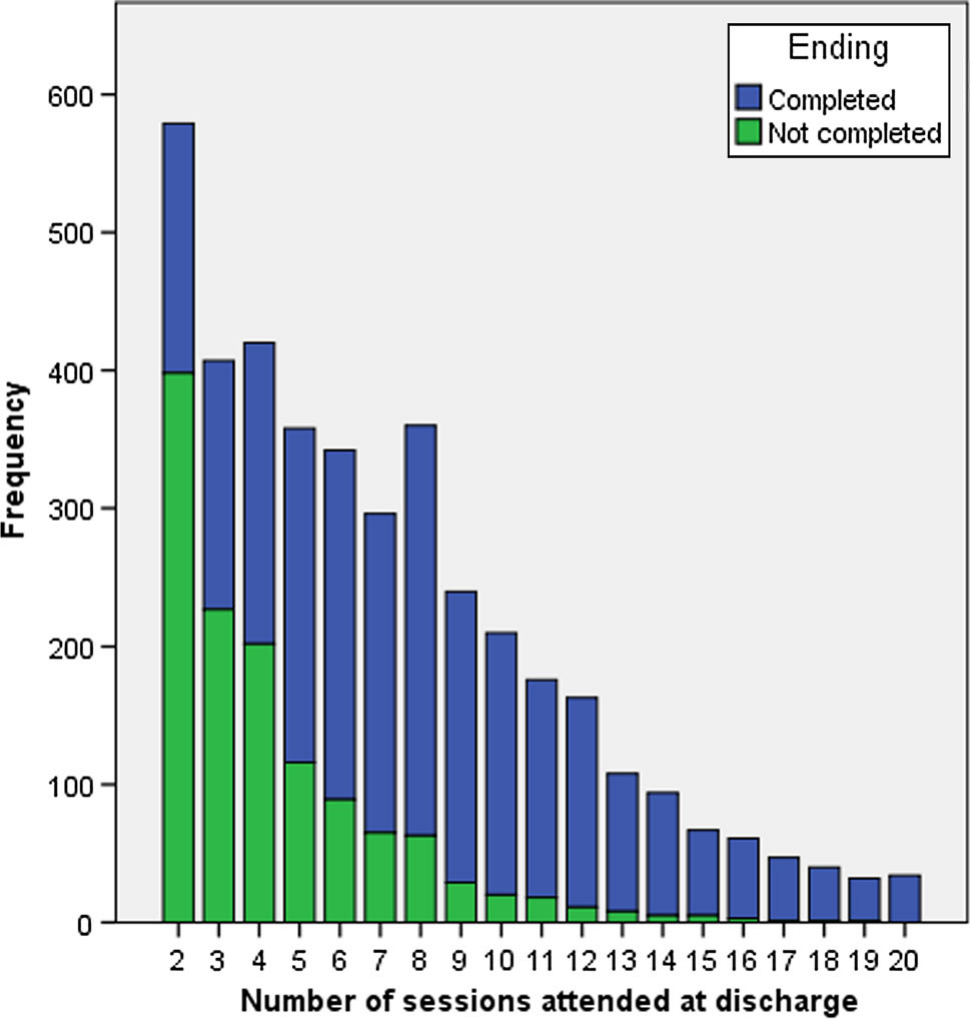
Frequencies overall and for completers and non-completers across the number of sessions attended

Overall, the mean (SD) number of sessions attended was 7.1 (4.29) with a median of 6 sessions (range 2–20) and a mode of two sessions. Figure 2 shows the frequencies for patients attending different numbers of sessions overall and for patients who completed or did not complete therapy.

Figure 2 shows that for non-completers, the modal number of sessions attended was two (31.5 %) and 86.9 % had stopped attending prior to session 8. The modal number of sessions attended by therapy completers was eight sessions (representing 10.7 % of all completers), with 47.1 % completing therapy prior to session eight and 36.3 % completing between sessions 8–12. The remaining 16.6 % completed therapy between sessions 13–20. Patients who did not complete therapy attended, on average, half as many sessions as those who completed therapy with a median (Range) of 4 (2–19) sessions, compared with 8 (2–20) sessions.

**Fig. 3:**
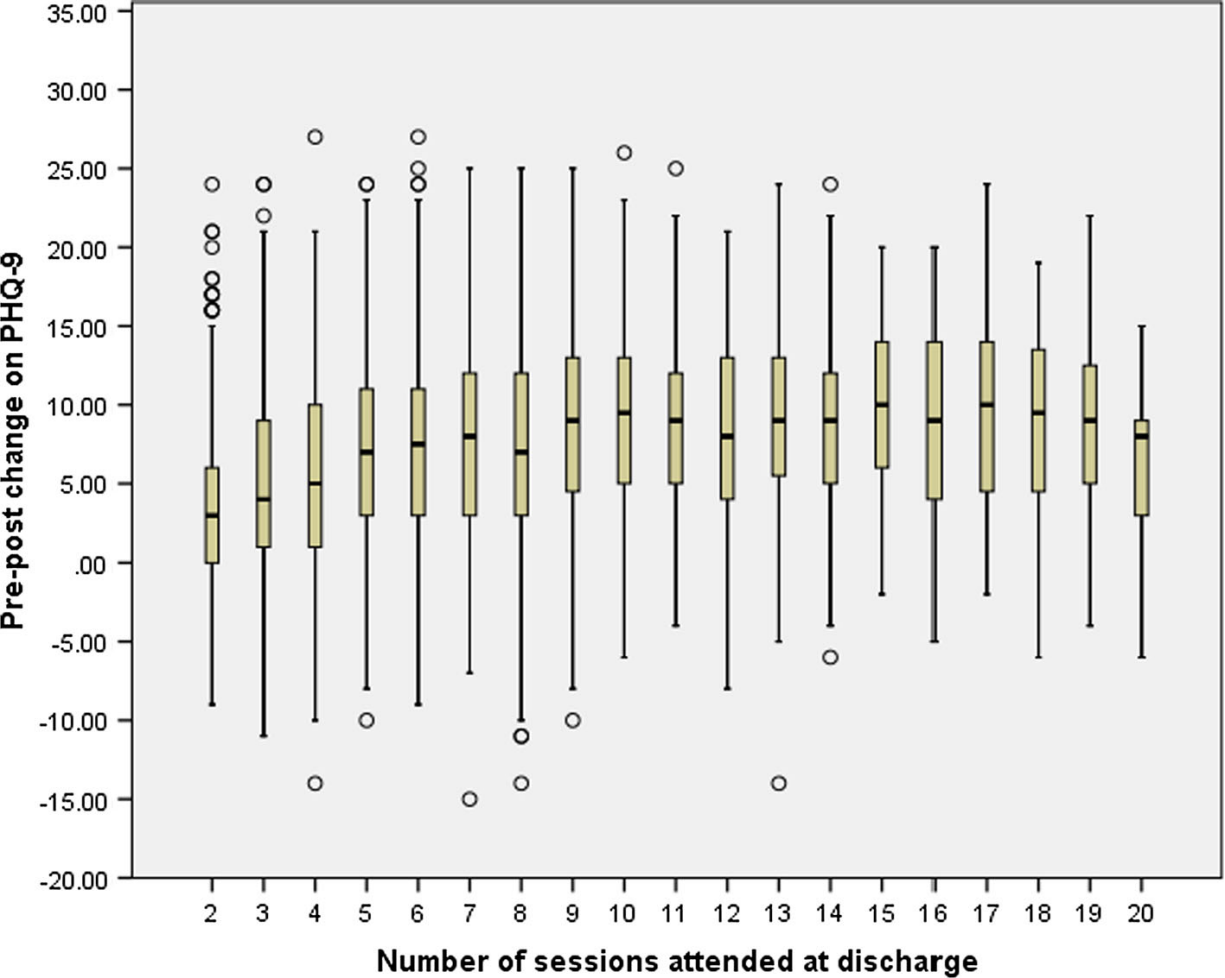
Boxplot of patient pre-post change on PHQ-9 across the number of sessions attended

The average amount of pre-post change in PHQ-9 scores, across the number of sessions patients attended is shown in the boxplot in Fig. 3. The median amount of change ranged from 3 points at 2 sessions, to 10 points at 15 and 17 sessions, although there does not appear to be a clear linear relationship between sessions and change. The amount of change increases by around a point per session up to 7 sessions, before levelling off at around 9 points of change thereafter.

The number of sessions attended by patients was compared between the three therapists groups identified in Fig. 1. Above average therapists provided, on average, one more session (Median: 7 sessions) than average therapists (Median: 6 sessions) and below average therapists (Median: 6 sessions). This one session difference was significant (K-W test: *p* < 0.001). However, the significant difference was only found for treatment completers (K-W test: *p* < 0.001), where above average therapists had a median of 9 sessions compared with 8 sessions for average and below average therapists. There was no significant difference between the three groups of therapists for treatment dropouts, where the median number of sessions for above and below average therapists was 4 sessions, compared to 3 sessions for average therapists (K-W test: *p* = 0.283).

The number of sessions attended (minus grand mean) was a significant predictor of outcome when added to the model, with a coefficient (SE) of +0.410 (0.051), indicating that attending more sessions generally improved outcomes, by about half a point on PHQ-9 for each additional session. However, the relationship of sessions to outcome was curvilinear and there was also a significant random slope. The relationship between sessions attended and outcome therefore varied across sessions and between therapists. A positive covariance between sessions and outcome (+0.238, SE: 0.079) shows that the variability between therapists increases as the number of sessions increases; that is, there is a ‘fanning-out’ of therapist regression lines. The therapist effect found of 5.8 % is for the mean number of sessions (7 sessions). However, this effect varies between 2 % at two sessions to around 40 % at 20 sessions, although estimates for higher numbers of sessions are derived from small samples.

**Fig. 4:**
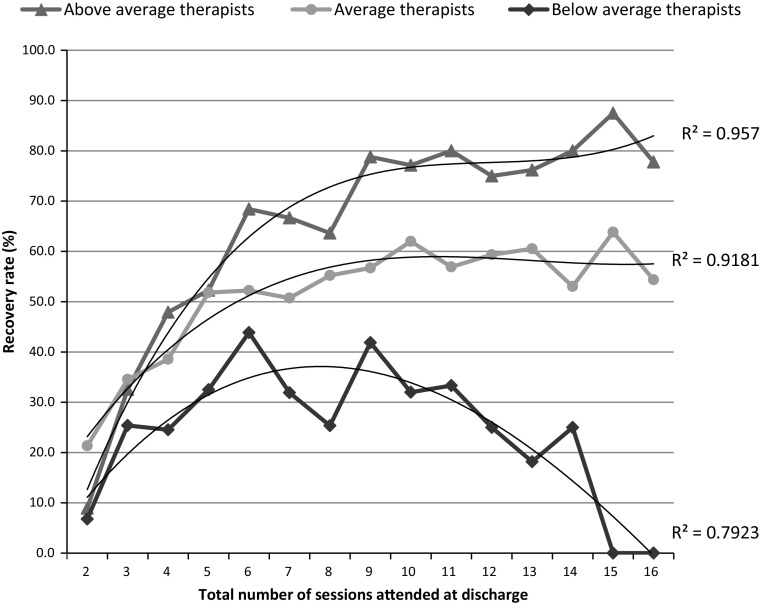
Statistical recovery rates for above average, average and below average therapists, for patients who attended 2–16 sessions. Lines of best fit are shown with R^2^ statistics

Figure 4 presents the recovery rates (statistically reliable and clinically significant improvement) for patients seen by the three groups of therapists identified in the caterpillar plot (Fig. 1), across the number of sessions that patients had attended by the end of therapy (i.e. their total dose at discharge). Because of the small number of patients who received more than 16 sessions (4.0 %, see Fig. 2), recovery rates for patients attending more than 16 sessions are not shown in Fig. 4. Only 15 (2.8 %) patients seen by below average therapists had more than 16 sessions, of whom 26.7 % recovered. For average therapists, 114 (3.9 %) had more than 16 sessions of whom 52.6 % recovered, while the number of patients attending more than 16 sessions with above average therapists was 24 (4.5 %) with 75.0 % recovered.

The lines of best fit in Fig. 4 show the curvilinear relationship between sessions attended and outcome as indicated by the model. The R^2^ statistics for each of these lines show they fit the data well, particularly for average and above average therapists. The model also indicated that there is less variability between therapists’ outcomes at fewer sessions, and that the variability increases as the sessions attended increases, the ‘fanning-out’ described by the model. The above average therapists’ recovery rates increase most rapidly as sessions increase from two to eight sessions while the increase is more gradual for average and particularly below average therapists. For patients who had eight sessions, the above average therapists were over twice as effective as below average therapists. After eight sessions, recovery rates begin to level out for average and above average therapists but decrease for the below average therapists. For patients who had twelve sessions, above average therapists were three times as effective as below average therapists.

### Therapy Endings

The 1262 patients (31.3 %) who did not complete therapy had significantly poorer outcomes compared to those who completed therapy. Their mean (SD) final PHQ-9 score was 15.5 (5.92) with a mean (SD) pre-post change of 2.9 (5.05) points. This compares with a final PHQ-9 score of 8.1 (6.10) and a pre-post change of 8.5 (6.07) points for therapy completers. Only 12.2 % of non-completers made statistically reliable and clinically significant improvement while 3.4 % reliably deteriorated, which compares with 60.2 and 1.1 % for completers (all *p* values <0.001).

**Fig. 5:**
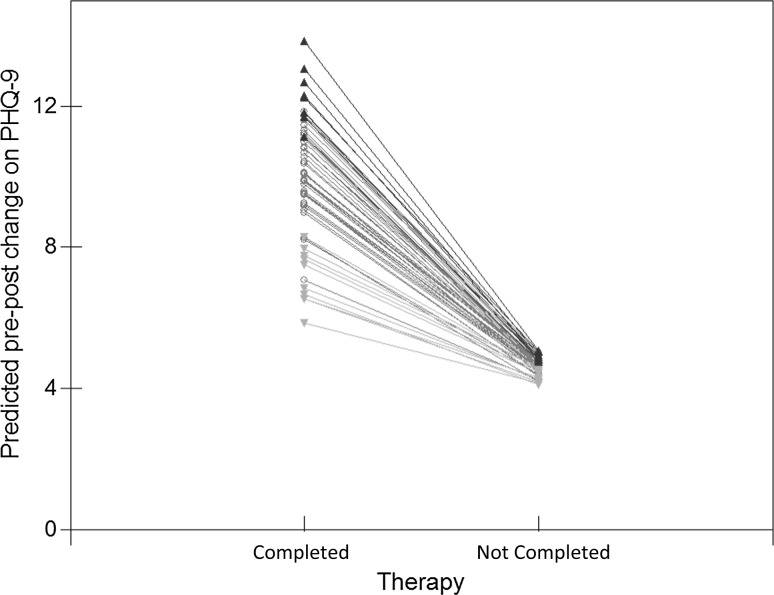
Predicted mean therapist pre-post change for patients who completed and did not complete therapy

Adding ‘therapy ending type’ to the multilevel model showed it to be a very strong predictor of outcome. Non-completion reduced the amount of PHQ-9 improvement by 6 points on average (coefficient: −5.996; SE: 0.283) compared to therapy completion. There was also a random slope indicating the relationship between ending type and outcome varied between therapists. The negative covariance suggests less therapist variability for patients who did not complete therapy. Modeling therapist effects for dropouts and completers separately, found no significant therapist effect for dropouts while the effect for completers was 11.2 %. This difference is shown in Fig. 5, which uses the model to plot predicted therapist mean pre-post change for completers and non-completers, controlling for case-mix and sessions. Therapists in the three different therapist groups are colour coded, grey for average, green for above average and red for below average. The plot shows the greater variability between therapists for patients who completed therapy than for patients who did not complete therapy, with the different therapist lines ‘fanning-in’.

For patients who completed therapy, the above average therapists’ outcomes are clearly distinct from those of below average therapists. The distinctions are less clear for patients who did not complete therapy. Therapists’ outcomes for non-completers correlated only weakly with their outcomes for completers (Pearson’s r: 0.32, *p* = 0.013). Table 2 describes the three therapist groups in terms of their patient outcomes for completers and non-completers.

**Table 2:** Comparison of completer and non-completer outcomes for patients seen by the three therapist groups

	Therapist group					
	Below average		Average		Above average	
	Completers	Non completers	Completers	Non completers	Completers	Non completers
N (%)	359 (66.1)	184 (33.9)	2021 (68.3)	937 (31.7)	392 (73.5)	141 (26.5)
Pre-post improvement mean (SD)	5.6 (6.22)	2.3 (4.76)	8.5 (5.89)	3.0 (5.16)	11.3 (5.57)	3.2 (4.64)
Recovery rate (%)	36.5	10.3	61.0	13.3	78.1	7.1

The differences in non-completion rates between therapist groups were significant, both between above average therapists and average therapists (*χ* ^2^(1) = 5.77, *p* = 0.016), and between above average and below average therapists (*χ* ^2^(1) = 7.05, *p* = 0.008) (see Table 2).

Comparing outcomes for therapy completers showed the differences in pre-post change between the three groups of therapists to be significant (ANCOVA: F (2,2768) = 91.44, *p* < 0.001) and the differences between pairs of therapist groups were also significant (all *p* values < 0.001). Similar results were obtained for recovery rates, (*χ* ^2^(2) = 137.03, *p* < 0.001).

However, for patients who did not complete therapy, the only significant difference was between the recovery rates for average and above average therapists (*χ* ^2^(1) = 4.37, *p* = 0.037). There were no significant differences on all other comparisons of outcomes with *p* values ranging from 0.08 to 0.994.

## Discussion

In this study of the variability of patient outcomes in naturalistic settings we sought to use practice-based evidence to complement the evidence-based research that informs policy, guidelines and service delivery. Using multilevel modeling to identify more and less effective therapists controlling for case-mix, we went on to consider therapist variability and outcomes in relation to three delivery factors: treatment modality, dosage and therapy ending. Our results indicate that differences between two evidence-based therapy models were less important for patient outcomes than the individual therapist they see, differences in dosage and in particular, whether the patient completed therapy or not. We also found that the effect that dose and ending type had on patient outcomes varied between therapists.

### Therapist Effect

The overall therapist effect found, of 5.8 %, although significant, is towards the lower end of the range of therapist effects found elsewhere (Crits-Christoph and Mintz 1991; Wampold and Brown 2005). However, larger effects were found where patients received more than the average number of sessions or completed therapy. Therapists’ recovery rates ranged from 16 to 76 % but the majority of therapists could not be considered significantly different from the average therapist after controlling for case-mix. However, the 13 % of therapists that were significantly more effective than average had recovery rates that were more than twice those of the 16 % of therapists identified as significantly less effective than average.

### Treatment Modality

We found an initial differential effect of therapy type, in favour of CBT, however the effect was small and clinically insignificant. This supports NICE depression guidelines (2016b) that, counseling should be available as an alternative to CBT and findings elsewhere that the therapy modality may have little effect when *bona fide* treatments of a specific condition are being compared (Luborsky and Singer 1975; Owen et al. 2015; Wampold et al. 2000). Moreover, we found that the small effect of therapy type disappeared when the differences between individual therapists in their relationships between dose and outcome and ending type and outcome were modelled.

### Therapeutic Dose

Our findings on the effect of dosage on outcomes develop further the evidence presented elsewhere, that the effect of dose varied between patients (Baldwin et al. 2009) and that there was variability in the amount of change per session achieved by different therapists (Okiishi et al. 2006). The current study found that the effect of dosage on patient outcomes varied *between* therapists, and that this variability increased as the dosage the patients received increased. This may be in part due to ‘more sessions’ being a reflection of the complexity and severity of a patient’s condition, given the limited number of sessions routinely offered, with additional sessions having to be agreed in clinical supervision. That there is greater variability between therapists for patients who are more difficult to treat would support findings reported previously using a different dataset (Saxon and Barkham 2012).

Generally, receiving more sessions improved outcomes, on average, by just under half a point on PHQ-9 for each additional session delivered. However, our results suggest that the ‘quality’ or ‘strength’ of the dose varied between therapists, with above average therapists yielding greater benefit per session compared to other therapists. Why some therapists can more rapidly improve their patient outcomes compared to other therapists and also maintain high recovery rates for patients receiving more sessions, needs to be studied further as it has important implications for effective and efficient therapy delivery.

### Therapy Ending

Any benefits from additional sessions can only be realised if patients do not drop out of therapy. Although the ending type and sessions attended are linked, with a greater frequency of non-completers at fewer sessions attended, our results show that of the two, type of ending is more important. Patients who complete a course of therapy improved, on average, by 6 more points as compared with patients who dropped out, while the benefit of each additional session was half a point on average. In terms of recovery rates, only 12 % of patients who dropped out of therapy recovered compared with 60 % for patients who completed therapy. This negative effect of therapy dropout is consistent with other findings (e.g. Cahill et al. 2003; Delgadillo et al. 2014).

There was less variability between therapist outcomes for patients who dropped out of therapy, compared to patients who completed. Our results indicate that although all therapists’ outcomes were negatively affected by dropout, there was a larger reduction in the recovery rate of therapy dropouts, relative to the rate for completers, for above average therapists compared to below average therapists. This was due to the above average therapists being considerably more effective with therapy completers. That above average therapists had more therapy completers also contributes to their relative effectiveness overall. Research to date suggests therapist skills in building the alliance and repairing ruptures seem to be strongly associated with therapy completion or not (Roos and Werbart 2013; Safran and Muran 2000).

### Limitations and Future Research

The naturalistic design of the study meant there was less control over certain aspects of therapeutic provision. However, this design means that the study is representative of the therapeutic provision routinely delivered in practice. Although we used a sample of patients above clinical cut-off on the PHQ-9 and focused on change in depression symptoms, controlling for anxiety, it was not known whether depression was the focus of the therapy as this is not recorded by the service and no formal diagnoses are made. This is a limitation of the current study, although reports indicate that depression and mixed anxiety and depression are by far the biggest reasons for referral to IAPT services (Health & Social Care Information Centre 2014).

The absence of other potential predictor and confounder variables such as a measure of therapeutic alliance or adherence was also a limitation. Treatment modality was the only therapist variable available and future research should investigate other therapist characteristics that may explain some of the variability between therapists. It would also be valuable for future research to examine sessional change trajectories—in particular, comparing CBT and counseling trajectories, and trajectories with more and less effective therapists. This was not possible with the current dataset.

Finally, the current study was carried out at a single IAPT site and results may not be generalizable to other types of therapy service. Future research should investigate therapist effects in relation to dose, treatment ending and patient outcomes in very large datasets from multiple sites, in order to consider any ‘site effects’. Where possible, these datasets should include variables such as sessional outcome measures, diagnosis and therapist factors and characteristics.

## Summary and Conclusions

We found significant variability between therapists’ outcomes after controlling for case-mix and that the effect on outcomes of sessions attended and patient drop-out, varied between therapists. More effective therapists were found to have fewer therapy dropouts and be more effective with therapy completers than less effective therapists. For therapy completers, more effective therapists delivered one more session on average than less effective therapists and were able to achieve greater change per session.

The current findings suggest that the two factors often given greater prominence in research, policy and delivery, namely therapy type and dose, may be less important for patient outcomes in services delivering evidence-based therapies, than the variability between therapists and maximizing the likelihood of patients completing a course of therapy. In order to inform therapist training, supervision and recruitment, future research should consider the features and characteristics of those therapists who are able to achieve greater improvement in their patients and more able to keep their patients in therapy to an agreed ending.

## Appendix

The multilevel model of pre-post change on PHQ-9, including case-mix variables

$$\begin{aligned} {\text{PPchange}}_{ij} & = \beta_{0j} + 0.503(0.030)({\text{FirstPHQ}}{\text{-}}{\text{gm}})_{ij} + - 0.098(0.026)({\text{FirstGAD}}{\text{-}}{\text{gm}})_{ij} + \\ & \quad - 2.577(0.209){\text{Unemployed}}\_1_{ij} + - 1.392(0.316){\text{NonWhite}}\_1_{ij} + \\ & \quad - 0.216(0.045)({\text{FirstPHQ}}{\text{-}}{\text{gm}}) \cdot {\text{Unemployed}}\_1_{ij} + e_{ij} \\ \end{aligned}$$

$$\beta_{0j} = 7.847(0.226) + u_{0j}$$

$$u_{0j} \sim N\left( {0,\sigma_{u0}^{2} } \right)\quad \sigma_{u0}^{2} = 2.117(0.499)$$

$$e_{ij} \sim N\left( {0,\sigma_{e}^{2} } \right)\quad \sigma_{e}^{2} = 34.641(0.777)$$

$$- 2*{\text{loglikelihood}} = 25{,}841.764\,(4034\,{\text{of}}\,4034\,{\text{cases}}\,{\text{in}}\,{\text{use}})$$
